# Impact of housing improvement and the socio-physical environment on the mental health of children’s carers: a cohort study in Australian Aboriginal communities

**DOI:** 10.1186/1471-2458-14-472

**Published:** 2014-05-19

**Authors:** Ross S Bailie, Matthew Stevens, Elizabeth L McDonald

**Affiliations:** 1 Menzies School of Health Research, Charles Darwin University, Tiwi, Darwin, Australia

**Keywords:** Housing, Mental health, Depression, Affect, Cohort, Child health, Indigenous, Negative life events, Stress

## Abstract

**Background:**

The mental health of carers is an important proximate factor in the causal web linking housing conditions to child health, as well as being important in its own right. Improved understanding of the nature of the relationships between housing conditions, carer mental health and child health outcomes is therefore important for informing the development of housing programs. This paper examines the relationship between the mental health of the carers of young children, housing conditions, and other key factors in the socio-physical environment.

**Methods:**

This analysis is part of a broader prospective cohort study of children living in Aboriginal communities in the Northern Territory (NT) of Australia at the time of major new community housing programs. Carer’s mental health was assessed using two validated scales: the Affect Balance scale and the Brief Screen for Depression. The quality of housing infrastructure was assessed through detailed surveys. Secondary explanatory variables included a range of socio-environmental factors, including validated measures of stressful life events. Hierarchical regression modelling was used to assess associations between outcome and explanatory variables at baseline, and associations between change in housing conditions and change in outcomes between baseline and follow-up.

**Results:**

There was no clear or consistent evidence of a causal relationship between the functional state of household infrastructure and the mental health of carers of young children. The strongest and most consistent associations with carer mental health were the measures of negative life events, with a dose–response relationship, and adjusted odds ratio of over 6 for carers in the highest stress exposure category at baseline, and consistent associations in the follow up analysis.

**Conclusions:**

The findings highlight the need for housing programs to be supported by social, behavioral and community-wide environmental programs if potential health gains are to be more fully realized, and for rigorous evaluation of such programs for the purpose of informing future housing initiatives.

## Background

The mental health of children’s carers and their ability to fulfil a parenting role are clearly important to the health and development of children [1,2]. Poor maternal mental health and depression have been identified as important underlying factors leading to poor child health and wellbeing outcomes internationally [1-3]. The wellbeing and mental health of children’s primary carers is a key factor in the relationship between biological risk factors, socio-economic conditions and their impact on child health and development [2,4].

Many of the risk factors for child health are to some extent inherent in the conditions in which people live, including their housing conditions. Improvement of the health and social conditions of disadvantaged communities has been a major objective of housing programs around the world [5-8]. There is accumulating research evidence that overall housing quality is positively correlated with psychological wellbeing [9-12], including specifically for women of childbearing age [13]. However, research into the relationships between housing and health has faced significant methodological challenges [10,14-16], and there continues to be a need for improved understanding of how housing programs can impact on the mental health of children’s carers for the purpose of informing the development of housing programs.

The Housing Improvement and Child Health (HICH) study examined the impact on child health of a major housing program in Aboriginal communities in the Northern Territory (NT) of Australia [17-21]. The study was a natural experiment that overcame a number of identified limitations of previous studies on housing and health, including comprehensive, objective and validated measurement of change in housing conditions, use of validated measures of health status, and assessment of moderating and mediating factors. Our previous reports on this study showed there was no clear evidence that the housing programs had resulted in reduction in the occurrence of common childhood infections in this study context. At a community level, the housing program had a marginal impact on the functional state of housing, and no measurable impact on household crowding or household hygienic conditions [18,19]. In the context of the NT program, likely contributing reasons for lack of demonstrable impact on child health include the complexity of mediating or intermediate variables in the causal web between housing conditions and child health, the limited scope of community housing support programs, and the lack of concurrent programs to address the general socio-physical environment.

An important and relatively distal intermediate variable in the causal web of housing improvement and child health is the mental health of the carers of young children. This is an important outcome in its own right, as well as being an intermediate step in improving child health outcomes. This paper therefore reports on an analysis of a) the association between housing conditions, other key factors in the socio-physical environment, and the mental health of the carers of young children, and b) the change in the mental health of carers following completion of the community housing program.

### Study setting

The NT contains several hundred remote Indigenous communities ranging in size from a single family group to 3,000 residents. Of the NT population of 200,000, Indigenous people make up approximately 30%, with over 70% of these people living in remote Indigenous communities [18]. Houses in remote communities range from modern design through to bricks and mortar, tin sheds and makeshift shelters. Houses are generally publicly owned and at the time of data collection (2003–2005) houses were frequently poorly maintained [22].

Indigenous people living in remote communities experience significantly poorer health and socioeconomic conditions than the general Australian population [23]. Young Indigenous children experience malnutrition and a high burden of common childhood infections that impacts on their healthy growth and development. Rates of notification and substantiation of child maltreatment for Indigenous children in the NT are nearly eight times that for their non-Indigenous counterparts, neglect being the most common identified cause of maltreatment [24].

The HICH study focussed on ten communities where there was the most substantial housing construction relative to the population and which reflected a wide geographic spread across the NT. Communities were between one and 500 km from the nearest regional town. The mean population for the ten communities was 730. The average of 11 people per house was markedly higher than the national average of 3.5 for Indigenous households and 2.6 for all Australians [25]. There was an average of 11 (range 7 – 15) houses constructed in each of the ten communities over the course of the study, with no concurrent renovation programs or hygiene promotion activities apart from routine maintenance programs that were operating in the communities prior to the new building programs. The study setting and intervention are described in more detail elsewhere [17,18,20].

## Methods

### Study design

The analysis presented here is part of the broader HICH study, which involved a prospective cohort of children aged 7 years or less who resided in the study communities at the time of the study. The conceptual framework for the study (http://www.biomedcentral.com/1471-2458/10/147/) [17] centres on the relationships between the functional state of household infrastructure and child health, and reflects a range of factors which may mediate or moderate this relationship. Data collection included: structured interviewer administered surveys of the main carer for each child in this age group and of the main householder; a systematic detailed survey of the functional state of the household infrastructure; a survey of the general community environment; and a semi-structured interview with a senior member of the community council or housing office. Table 1 provides an overview of the primary and secondary explanatory variables and how these map to the study conceptual framework.

**Table 1:** **Primary and secondary explanatory variables and how these map to the study conceptual framework *(***http://www.biomedcentral.com/1471-2458/10/147***)***

**Primary explanatory variables**^**1**^			
***Household infrastructure function.*** Two measures: Number of HLP components required for healthy living practices failed; Overall Surveyor Function Score			
**Secondary explanatory variables**			
***Carer socio-demographic***	***Child health and health behaviour and hygiene***	***Carer socioeconomic status*** ***and financial stress***^**3**^	***Psychosocial and health***
Community of residence^2^	*Child Health*^4^	Carer highest level of schooling	Other people from tribal group live in community^2^
*Carer socio-demographic*^3^	Number of illnesses in carer’s children in past two weeks		Frequency of visits to traditional land^3^
• sex		Carer labour force status	Number of people get help from if has serious worries^2,3^
• age			Carer self-reported health^7^
• cohabitation with spouse	*Health behaviour, hygiene and day care*	Household material wealth	*Carer Negative Life Events*^2,3^
			• Worried about someone sick/disabled
Carer relationship to householder		Householder holds important position in community	• Know someone who had a bad accident
Time that carer has lived in the house	Broom, mop and bucket in house^4^		• Death of family member or close friend
Carer mobility between communities (lived in other community for more than 4 weeks)	Soap in bathroom, kitchen^5^		
Number of children cared for	Household hygienic condition (surveyor condition score)^6^	*Financial security*	• Member of family in jail or sent to jail
• aged less than one year		• Ran out of money in last 2 weeks	
• aged 1–3 years	Number of children in day-care^3^		• Too many people living in one house
• aged 4–7 years		• Ran out of money in last year	
• aged 8 to 15 years			• Worried about divorce/separation
Number of adults in house		• Number of things did to get money if ran out	
			• Not able to get a job
		• Raise $2000 in a week for emergency	• Lost their job/sacked
			• Alcohol or drug problems
			• Seeing fights and people beaten up
			• Someone being abused or victim of violent crime
			• Trouble with police
			• Gambling problems
			• Racism

***Community level influences:*** crowding, general condition of housing stock, community environmental conditions and infrastructure, availability of community facilities, community safety (community level concerns based on data items from the Negative Life Events Scale: concerns about fights or violence, abuse or trouble with the police).

***Variables mapped to conceptual framework*** *(*http://www.biomedcentral.com/1471-2458/10/147*)*.

^1^Facilities for Healthy Living Practices; ^2^Community and Neighbourhood Influences; ^3^Household Composition and Process; ^4^Child Health; ^5^Software; ^6^Condition of Household Environment; ^7^Carer Health.

Timing and other plans for the building programs were staggered over the period covered by the study, and were subject to change for a range of reasons beyond the control of the study investigators - sometimes with relatively short notice. Timing of data collection for the study was adjusted around changes to the building programs, within resource and timing constraints imposed by research funding. Baseline data collection was completed on average six months (range 1–18) prior to occupation of new houses in each community. Follow-up interviews and surveys were completed on average 10 months (range 7–12) after occupation of new houses. Baseline data were analysed to identify associations between carer mental status, housing conditions and a range of socio-demographic and other health related variables. Follow-up data were analysed for evidence on the causal direction between carer mental health status and variables for which there are significant independent associations at baseline. Additional details about the conceptual framework and more general methods used in the HICH Study are described in detail in previous publications [17-20].

### Measures of carer’s mental health

Two scales were used to assess carer’s mental health, the Affect Balance (AB) scale [26] and the Brief Screen for Depression (BSD) [27].

The AB scale was used previously in Indigenous populations in North America [28]. Use of the scale in the HICH study population revealed a Cronbach’s Alpha for positive and negative dimensions respectively of 0.66 and 0.58, and overall 0.59. The positive affect scale showed a poor spread across possible scores with more than 70% of carers having scores at the most positive end of the scale. Exploratory analysis indicated that very few variables showed a significant association with positive affect. We have therefore focused our analysis on the negative affect scale, with a high negative affect defined as a score of four or more (which equated approximately to the most negative quintile of respondents). To create a measure of change the score at follow-up was subtracted from the score at baseline, with the variable having a potential range from -4 to 4. A positive change in this variable indicates an improvement in carer affect.

The BSD consists of four questions, with three using a Likert scale of one to ten and the other a scale of one to five. In calculating the total score, scores for individual questions are added, with the score for the 5-point Likert scale doubled, thereby giving a possible score of between five and forty, a score of ≥25 indicating a high risk of depression. Cronbach’s Alpha for internal consistency was 0.54. For the purpose of measuring change over time, the score at follow-up was subtracted from the score at baseline, with a potential range of -30 to 30. A positive score indicates an improvement in carer mental health.

### Primary explanatory variables

The survey of houses involved inspection by experienced surveyors using a standard checklist of the functional state of infrastructure required for the residents to carry out a set of ‘Healthy Living Practices’ (HLPs) [29,30]. Details of the HLPs and the specific items of infrastructure required for each HLP are presented in Table 1 of a previously published paper (see http://www.biomedcentral.com/1471-2458/10/147/). For the first method, surveyors assigned a score of 1 (good) to 7 (poor) for each of the thirteen HLPs, and an overall house score. Visual inspection and testing (as required) were used to determine the score, and this method is referred to as the Surveyor Function Score (SFS). For the second method, sets of individual infrastructure items were matched to eight HLPs (this pass/fail method was not suited to five of the HLPs). If all infrastructure items in the set that make up the HLP were fully functioning (or had only minor problems), then the house was scored as “pass” on that HLP. An overall house score was derived by adding the number of HLPs “failed” (scores of 0 (good) to 8 (poor)). This method is referred to as the Failed Healthy Living Practice (FHLP) score. The survey process, the two methods used to score health-related infrastructure, and the repeatability of the methods have been described in more detail elsewhere [17,20].

For the follow-up analyses, the change in the SFS for each HLP and the overall house SFS was derived by subtracting the score at baseline from the score at follow-up, which was then categorised into three groups: 1) limited or no change in score (i.e. one point or less); 2) deterioration of two or more points; and 3) improvement of two or more points. For the binary FHLP measures, we were interested in the effect of an improvement, so from the four possible groupings of the pass/fail dichotomy (pass to pass, pass to fail, fail to pass, fail to fail), a binary variable was derived. Categories for this variable were: 1) a carer living in a house at baseline that failed the particular HLP to living in a house at follow-up that passed (i.e. improvement); and 2) all other possibilities (i.e. no improvement). A change in the overall number of HLPs failed was constructed in the same way as the change in SFS, and grouped using the same cut-points, with the reference category being limited or no change in the number of HLPs failed.

### Secondary explanatory variables

Carer, householder and housing-related variables were divided into five groups: 1) the primary explanatory variables (SFSs and FHLPs); 2) socio-demographic; 3) health-related behaviour, hygiene, and children’s health,; 4) socio-economic status and financial stress; and 5) psychosocial factors and self-reported health.

Carer socio-demographic variables were community of residence, age, sex, spousal cohabitation, relationship to the householder, time living in house, mobility (frequency of movement between houses), number of children in care (by age), and number of adults in the house.

Health-related behaviour and hygiene variables included presence of a broom, mop and bucket (all/missing one or more); presence of soap (yes/no); number of children in day-care; and thirteen Surveyor Condition Scores (SCS), each one relating to the hygienic state of the house for the same thirteen HLPs referred to above. These were rated on a 7-point Likert scale similar to that used for the SFS and have been described in detail elsewhere [17]. Scores were dichotomised for the analyses presented in this paper.

Information collected on child health was aggregated to the carer level (see below under Data and statistical analysis), so these variables reflect the health of all children for which each carer was responsible. Child health data were obtained by asking carers whether their child had any of five common childhood illnesses in the 2 weeks preceding the survey. Specifically, they were asked about skin infections (without scabies), scabies, diarrhoea and vomiting, respiratory and ear infections. From the five illnesses a variable was derived indicating total number of illnesses amongst all the children for whom the carer was responsible.

Carer socioeconomic status and financial stress variables included highest level of schooling, labour force status, material wealth (working telephone and fridge), householder status in community (whether the main householder holds one or more important positions in the community), financial security (a. whether ran out of money in last two weeks (or in last year), b. number of things did to get money if ran out, and c. perceived ability to raise $2000 in an emergency).

The psychosocial factors and self reported health variables included: number of other people from their tribal (clan) group in the community; frequency of visits to traditional land; number of people they could get help from outside the house if they have serious worries; self-reported health; and items from the Negative Life Events Scale. The Negative Life Events Scale (NLES) measured exposure to thirteen ‘negative life events’ or ‘stressors’ [31]. It was developed by the Australian Bureau of Statistics (ABS) in conjunction with peak Indigenous bodies as a measure of emotional and social well being for use in national Indigenous and general population social and health surveys [32]. Carers answered yes or no to the question of whether they or anyone in the house had experienced each of thirteen ‘stressors’ over the previous 12 month period. The psychometric properties of this scale in this study population have been reported elsewhere [21]. In order to examine associations between mental health and overall exposure to negative life events we created a composite NLES variable by adding the total number of NLES items reported and divided this score into quartiles.

For the follow-up analysis, we created variables that reflected a change between baseline and follow-up. For example, for NLES items, the new variable indicated that the carer went from reporting a stressor at baseline to not reporting it at follow-up.

### Statistical analyses

For the purpose of the analysis presented in this paper all variables were defined at the individual carer level. Thus, for variables that were measured at the child level (for example, number of illnesses), where a carer had responsibility for more than one child the data were aggregated to carer level. For variables measured at the household level (for example, household SCS), the same data were used for each carer in the house (where there was more than one carer in the house). Exploratory analysis was carried out and cut-points determined for variables to ensure adequate distribution across categories for subsequent statistical analyses.

#### Cross-sectional analysis

The two outcome variables for analysis (high negative affect and high risk of depression) are dichotomous and therefore suited to logistic regression modelling. The following steps were carried out for each outcome. We used a hierarchical approach to the statistical analysis for each of the outcome variables, with bivariate associations initially identified between the outcome variable and the explanatory variables within each of the explanatory variable domains as defined above. Within each domain, explanatory variables that showed moderate evidence (p ≤ 0.10) of an association with the outcome variable were then entered simultaneously into a model and backward selection applied with removal set at p>0.05. Variables that remained significant (p ≤ 0.05) in the models for each domain were then all entered simultaneously into a model and backward selection again applied, with removal at p>0.05.

For the psychosocial variables, NLES items were entered into models separately from the composite NLES measures described above. If individual items and composite NLES variables were significant, then the previously described step was carried out separately for individual items and composite NLES measures.

Plausible first-order interactions were tested in final models and reported where significant. Preliminary and cross-sectional analyses indicated that the Community ID was exerting a strong effect and was therefore treated separately in the analysis and was not included in the socio-demographic domain for the analyses.

Once final models were developed using the process described above, the community ID variable was entered separately into each model to assess whether there was an independent significant influence of community. Variables that became non-significant (p > 0.05) after inclusion of the community ID variable were then removed.

For each outcome, either one or two models are presented, depending on whether the community variable remained significant in the model. This approach is consistent with that which we used for the analysis of child health outcomes previously reported [17].

#### Follow-up analysis

We used a stepped hierarchical approach (similar to that for the cross sectional analysis as described above) using linear regression modelling to explore associations between change in explanatory variables and change in carer mental health outcome measures (change in negative affect score and change in BSD score). For explanatory variables that were not subject to change (or were less subject to variation over time) - such as carer age, sex, and education - baseline values were used.

#### Understanding community level influences

Data from the survey of the general community environment and semi-structured interviews with key informants were used to characterise communities in terms of general level of crowding, general condition of housing stock, environmental conditions and infrastructure, and availability of community facilities. Data items relating to community safety (concerns about fights or violence, abuse or trouble with the police) from the NLES that was used as part of the interviews with carers were also aggregated to create community level variables. Communities were ranked according to these community level variables. The ranking was used to determine distinguishing features of communities where the community ID showed an independent significant association with the outcome variables used in the cross sectional and follow-up analysis.

All analyses were carried out using Stata Statistical Software Version 10.1. Standard errors (and confidence intervals) in logistic and linear regression models were adjusted for clustering of carers in communities and dwellings using the Huber-White sandwich estimator method. This type of adjustment has been shown to be robust and to produce unbiased estimates for cluster-correlated data regardless of the setting [33].

Ethics approval was obtained from the Human Research Ethics Committees in the Top End and Central regions of the NT and formal agreements to participate were signed by peak organisations in each of the study communities. Informed consent to participate in the study was obtained from householders and children’s carers prior to the conduct of household surveys and interviews.

## Results

### Cross sectional analysis

#### Unadjusted associations

**Figure 1:**
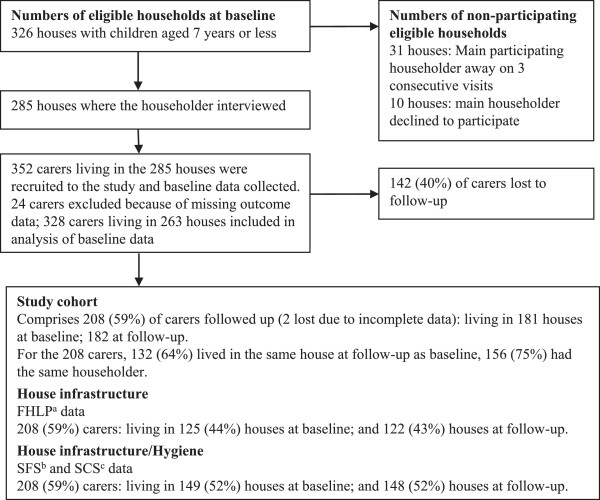
Primary exposure variables: crowding, house infrastructure and hygiene data at baseline and follow-up.

Of 352 carers who participated in the baseline survey, 24 (7%) were excluded due to missing data in either of the two outcome variables (Figure 1). The data for a total of 328 carers were therefore included in the analysis of baseline data. These carers lived in 263 different houses.

Using the BSD and the AB negative affect scale as described above, 16% of carers were categorised as being at high risk of depression, and 23% as having high negative affect (Table 2).

**Table 2:** Unadjusted odds ratios (95% confidence interval) between functional state of household infrastructure and carer negative affect and risk of depression at baseline

**Primary explanatory variables**			**High negative affect**		**High risk of depression**	
	**Missing**	**Carers**				
	**n (%)**	**n (%)**	**n (%)**^**1**^	**OR (95% CI)**	**n (%)**^**1**^	**OR (95% CI)**
**All carers**		**328 (100)**	**75 (22.9)**	-	**53 (16.2)**	**-**
***Number of HLP components failed***^***2***^						
0-4 (better function)	44 (13.4)	94 (33.1)	25 (26.6)	1.0	8 (8.5)	1.0
5-8 (poorer function)		190 (66.9)	39 (20.5)	0.71 (0.42-1.22)	40 (21.1)	**2.87 (1.18-6.94)**
***Overall Surveyor Function Score***^***2***^						
Better (scores 1–2)	17 (5.2)	88 (28.3)	20 (22.7)	1.0	13 (14.4)	1.0
Worse (scores 3–7)		223 (71.7)	49 (22.0)	0.96 (0.54-1.69)	38 (17.2)	1.23 (0.59-2.55)

^1^Number and percentage of carers classified as having high negative affect or being at high risk of depression.

^2^Functional state of infrastructure components required to conduct Healthy Living Practices (HLPs) were observed (and tested where appropriate) by the surveyor.

NOTE: Bold font indicates the variable was significant at p ≤ 0.05.

Unadjusted analysis of associations between functional state of household infrastructure showed the odds ratios for carers being at high risk of depression was almost three times higher for those living in houses failing five or more of the HLP components, compared with those living in houses failing less than five (OR 2.87, CI 1.18-6.94). There were no significant associations between either of the measures of functional state of household infrastructure (SFS and HLPs) and high negative affect among carers (Table 2).

A number of the secondary explanatory variables were shown to be associated with high negative affect and high risk of depression in the unadjusted analysis (Additional files 1, 2, 3 and 4). Carers in some communities were significantly more likely to have high negative affect (odds ratio nearly eight times higher in one community and over three times higher in another). In contrast, the odds ratio for carers being at high risk of depression did not differ significantly between communities.

Reporting of a high number of illnesses among the children for whom the carer was responsible showed an odds ratio of more than three for both high negative affect and high risk of depression. Other secondary explanatory variables that showed an odds ratio of more than three for high negative affect were all related to the NLES (specifically a serious accident, loss of a job, trouble with the police, gambling problems, and multiple negative life events). Similarly, loss of a job, death of a family member or close friend and multiple negative life events showed an odds ratio of more than three for high risk of depression.

#### Multivariate models

There were no significant associations between the housing infrastructure variables and carer negative affect in the baseline survey. The housing infrastructure variables were therefore dropped from the multivariate models for this outcome. The most parsimonious multivariate model for high negative affect among carers was derived using the composite NLES measure (this model had an adjusted R^2^ of 18.9%) (Table 3). There was a significant association between number of NLES with high negative affect, with an increasingly strong association with increase in number of reported negative life events (6 to 8 items - OR 3.50 (CI 1.31-9.14), 9 to 13 items - OR 6.07 (CI 2.39-15.5)).

**Table 3:** Multivariable adjusted logistic regression models for high negative affect (n = 316) and high risk of depression (n = 266) among carers at baseline

**Explanatory variable**	**High negative affect OR (95% CI)**	**High risk of depression OR (95% CI)**
***Number of HLPs not met***		
0-4	ns	1.0
5-8	ns	**2.23 (0.98-5.07)**
***Community ID***		
1	1.0	ns
2	1.82 (0.47-7.05)	ns
3	**9.84 (3.10-31.2)**	ns
4	2.37 (0.64-8.84)	ns
5	**3.29 (1.22-8.90)**	ns
6	1.28 (0.14-11.7)	ns
7	1.21 (0.33-4.44)	ns
8	2.41 (0.91-6.42)	ns
9	2.49 (0.66-9.37)	ns
10	1.17 (0.20-6.73)	ns
***Number of children cared for aged 8–15 years***		
None	1.0	ns
One	0.45 (0.20-1.02)	ns
Two or more	**0.26 (0.12-0.58)**	ns
***Number of children in day-care***		
No	1.0	ns
One to three	**2.85 (1.17-6.91)**	ns
***Carer ran out of money in the last 2 weeks***		
No	1.0	ns
Yes	**2.64 (1.36-5.14)**	ns
***Carer NLES quartiles (number of stressors reported)***		
0 to 3	1.0	ns
4 to 5	2.38 (0.87-6.52)	ns
6 to 8	**3.50 (1.34-9.14)**	ns
9 to 13	**6.07 (2.39-15.5)**	ns
***Sacked or made redundant***^***1***^		
No	ns	1.0
Yes	ns	**4.43 (1.24-15.9)**
Death of a family member/close friend in the last year^1^		
No	na	1.0
Yes	na	**3.67 (1.49-9.06)**
***Adjusted R***^***2***^	*18.9%*	*7.5%*

^1^Carer reported for themselves or someone in the house in the past year (NLES item).

Bold font indicates the variable was significant at p ≤ 0.05; ns – not significant; na – not applicable.

Other carer level variables that were associated with high negative affect were: running out of money in the last two weeks (OR 2.64, CI 1.36-5.14); and having at least one child in day-care (OR 2.85, 1.17 to 6.91). Carers who cared for more than two children aged 8–15 years had significantly reduced odds of high negative affect (OR 0.28, CI 0.13-0.57).

The multivariate model for high negative affect identified two communities (Community 2 and Community 5) where carers had significantly higher odds for this outcome. Both of these communities were inland communities in the monsoon tropics (i.e. not coastal, not desert communities). Both were of medium size (400–500 residents) compared to the other ten communities. One had no outstations (small outlying settlements); the other had about 4 outstations.

Analysis of the community survey data showed these two communities to be among the three worst communities in relation to proportion of carers reporting concerns about fights or violence, abuse, or trouble with the police, and in terms of overall functional state of housing. Both communities were amongst the most isolated of the ten communities in terms of distance to major service centres (a centre with facilities such as a high school, hospital), and were amongst the poorest of the ten communities in terms of the community environmental health conditions and environmental health infrastructure (including relatively poor environmental health staffing, having an unfenced tip within one km of the community, relatively less frequent removal of household rubbish, no functioning public toilets, relatively frequent sewage overflows or leakages, relatively frequent ponding of flood waters affecting homes). Community 5 also experienced relatively frequent power interruptions, relatively poor community facilities (such as a hall, arts centre, women’s centre, sports facilities), and higher level of household crowding than most other communities.

The multivariate model for high risk of depression showed a marginally non-significant association between poor household infrastructure function and this outcome (OR 2.23, CI 0.98-5.07). The most parsimonious model for high risk of depression was derived using individual NLES items (adjusted R^2^ of 8.5%). This model also showed that carers who reported worry over the death of a family member or close friend (OR 3.68, CI 1.49-9.06) were more likely to be at high risk of depression. Carers living in a house where someone had been sacked/redundant from a job were also more likely to be at high risk of depression (OR 4.43, CI 1.23-15.9).

### Follow-up analysis

Of the 328 carers included in the analysis of baseline data, we were able to obtain follow-up data on one or other of the outcome variables for 208 carers (63%) (Figure 1). Data on negative affect were available at both baseline and follow-up for 166 carers (51%), and on risk of depression for 151 carers (46%).

#### Unadjusted associations

Unadjusted associations between primary and secondary explanatory variables showing a significant association with change (∆) in carer negative affect and risk of depression are shown in Additional file 5. An improvement in household infrastructure (as measured by the FHLP score) was associated with a significant improvement in carer negative affect, but there was no association between improvement in household infrastructure and risk of depression (Additional file 5).

The secondary explanatory variables with the strongest association with change in negative affect and in risk of depression (as reflected by relatively large and significant *β* coefficients) included community ID, where carers living in Community 5 showed a significant improvement in both measures of carer mental health. Change from knowing someone who had a serious accident within the past 12 months at baseline to not knowing someone in this situation at follow-up also showed relatively strong associations with improved negative affect and reduced risk of depression.

Other secondary explanatory variables that showed relatively strong associations with change in negative affect included change in child day care attendance. Change from having a child in day care at baseline to not having a child in day care at follow-up was associated with improved negative affect. Young age (<20 years) at baseline was strongly associated with increased risk of depression at follow-up.

#### Multivariate models

The multivariate analysis of follow-up data did not show significant association between improved household infrastructure function and carer psychosocial outcomes. Two multivariate linear regression models are presented for change in carer negative affect - model 2 includes the community ID variable, model 1 does not (Table 4). Model 2 had a higher adjusted R^2^ than model 1 (35.1% compared to 22.6%). Only one multivariate linear regression model is reported for change in carer risk of depression (adjusted R^2^ of 22.1%), as the community variable did not show a significant unadjusted association.

**Table 4:** Multivariate adjusted linear regression: change in carer negative affect (n = 159) and change in carer risk of depression (n = 133) between baseline and follow-up

**Explanatory variable**	_**Model 1**_	_**Model 2**_	_**Model 3**_
	**∆ Negative affect**^**1 **^ ***β*** **(95% CI)**	**∆ Negative affect**^**1 **^ ***β*** **(95% CI)**	**∆ Risk of depression**^**2 **^ ***β*** **(95% CI)**
***Community ID***			
1	ni	-0.28 (-1.13, 0.58)	ns
2	ni	-0.74 (-1.52, 0.03)	ns
3	ni	0.17 (-0.50, 0.84)	ns
4	ni	0.81 (-0.31, 1.92)	ns
5	ni	**1.78 (1.00, 2.56)**	ns
6	ni	-0.15 (-1.13, 0.84)	ns
7	ni	-0.82 (-1.65, 0.01)	ns
8 (median change in negative affect)	ni	0.0	ns
9	ni	0.35 (-1.06, 1.77)	ns
10	ni	1.26 (-0.21, 2.72)	ns
***Carer age at baseline***			
<20 yrs	ns	ns	**-9.70 (-15.58, -3.83)**
20-34 yrs	ns	ns	0.0
35 + yrs	ns	ns	0.76 (-2.28, 3.80)
***Change in carer ran out of money last year***			
Other	0.0	ns	ns
From had money to ran out of money last year	**-0.62 (-1.18, -0.05)**	ns	ns
***Change in child day care attendance***			
No change	ns	ns	0.0
Change from No to Yes	ns	ns	**-4.03 (-6.37, -1.68)**
Change from Yes to No	ns	ns	-1.66 (-9.55, 6.23)
***∆ in number of people carer could get help from not in house***			
Other	0.0	0.0	ns
From none to able to get help from one or more	**0.86 (0.26, 1.45)**	**0.65 (0.05, 1.26)**	ns
***Change in carer’s has clan/kin in community***			
Other	ns	ns	0.0
Had clan/kin in community to did not	ns	ns	**-3.55 (-5.46, -1.64)**
***NLES: ∆ in knowing someone who had a serious accident***			
Other	0.0	0.0	0.0
From yes to no	**1.18 (0.59, 1.78)**	**0.98 (0.48, 1.47)**	**9.38 (3.44, 15.31)**
***NLES: ∆ in knowing someone sent to jail***			
Other	0.0	ns	ns
From yes to no	**1.13 (0.53, 1.73)**	ns	ns
***Adjusted R***^***2***^	*22.6%*	*35.1%*	*22.1%*

1 Carer Negative affect balance (range: -4 to 4); 2 Carer Risk of depression (range: -30 to 30).

A positive Beta coefficient indicates the variable is associated with an improvement in mental health. Bold font indicates the variable was significant at p ≤ 0.05; ni – not applicable; ns – not significant.

Among the secondary explanatory variables, carers reporting a change from knowing someone in a serious accident in the previous year at baseline to not knowing someone in this situation at follow-up showed independent significant association with improvement in negative affect in both model 1 and model 2 (*β* = 1.18, CI 0.59 to 1.78; and *β* = 1.13, CI 0.53 to 1.73, respectively) and with improvement in risk of depression (*β* = 9.38, CI 3.44 to 15.31).

Carers who reported a change from not having someone from outside their house they could get help from at baseline to having one or more people they could get help at follow-up showed independent significant association with improvement in negative affect in both model 1 and model 2 (*β* = 0.86, CI 0.26 to 1.45; and *β* = 0.65, CI 0.05 to 1.26, respectively).

Two other secondary explanatory variables showed statistically significant associations with improvement in negative affect in model 1, but not in model 2 where community ID was included. In model 1 carers who reported that they had not run out of money in the year prior to baseline and then did report this stressor in the year prior to follow-up showed a deterioration in negative affect (*β* = -0.62, CI -1.18 to -0.05) in model 1, while carers who reported knowing someone sent to jail in the year prior to baseline and then did not report this stressor in the year prior to follow-up showed an improvement in negative affect (*β* = 1.13, CI 0.53 to 1.73). In model 2 carers living in Community 5 showed a significant association with improvement in negative affect. Community ID did not remain significant in the linear regression model for change in risk of depression.

Change in risk of depression was significantly independently associated with three other secondary explanatory variables in addition to the variable reported above on knowing someone involved in a serious accident: carers less than 20 years at baseline showed a significant increase in risk of depression at follow-up (*β* = -9.70, CI -15.58 to -3.83), as did carers reporting not having a child in day care at baseline to the child being in day care at follow-up (*β* = -4.03, CI -6.37 to -1.68), and those who reported having clan or kin in their community at baseline to not having clan or kin in their community at follow-up (*β* = -3.55, CI -5.46 to -1.64).

#### Consistency of cross-sectional and follow-up multivariate models

Variables that showed an independent association with the outcomes in multivariate models for the baseline cross-sectional and the follow-up analyses (Tables 3 and 4) were community ID and the measure of financial difficulty (carer ran out of money in the last two weeks (baseline analysis), carer ran out of money in the last year (follow-up analysis)). Variables relating to the NLES were significant in both the cross-sectional and follow-up analyses, although they were not exactly the same variables.

Community 5 is one of the two communities that showed a significant independent association with high negative affect at baseline, and also shows a significant independent association with improved negative affect in the follow-up analysis. The number of new houses in this community was higher than the median number of new houses for the ten communities (proportional to community size). While community 5 had the second highest level of household crowding at baseline, it had the second largest reduction in household crowding at follow-up. From being among the three worst communities at baseline in relation to proportion of carers reporting concerns about fights or violence, abuse, or trouble with the police, at follow-up Community 5 was amongst the three communities were carers were least likely to report such concerns.

## Discussion

This study does not show clear or consistent evidence of a causal relationship between the functional state of household infrastructure and the mental health of carers of young children in this study context. While there was a relatively strong independent association between functional state of household infrastructure and risk of depression at baseline (OR 3.26; CI 1.33-7.98), this association was not evident in the analysis of the follow-up data. For negative AB, there was a significant association between improvement in household infrastructure function and improvement in affect in the unadjusted analysis, but not in the adjusted analysis. The significant associations between housing infrastructure variables and the measures of carer mental health that were found are therefore likely to be due to confounding or chance association.

The variables that show the strongest and most consistent associations with the measures of carer mental health used in this study are the measures of negative life events. Positive associations were evident in the cross-sectional and follow-up analysis for both risk of depression and for negative affect. The findings point to the significant influence of high rates of illness, injury and early death on mental health. Other community level factors also appear to have an important influence on negative affect, including poor community safety (or frequency of violent events), general condition of housing stock and levels of crowding in the community, and community isolation.

The findings regarding the significance of community level variables are consistent with our previous reported analysis of the community level impact of the housing program on crowding, infrastructure function and hygiene [18], and on child health outcomes [19]. High levels of household crowding have commonly been reported to be associated with poor mental health [10,11] and psychosocial stress [9,12], but the nature of these associations is far from clear. There is substantial research pointing to the relative importance of area level compared to individual or household level effects. Neighbourhood characteristics are reported to be associated with various health outcomes including depressive symptoms [34,35]. Canadian research on the mechanisms through which the effects of neighbourhood socioeconomic conditions impact on young children’s verbal and behavioural outcomes found that neighbourhood disadvantage manifested its effect via lower neighbourhood cohesion [36]. This was associated with maternal depression and family dysfunction and lead to less consistent, less stimulating, and more punitive parenting behaviours, and ultimately, poorer child outcomes. Lower neighbourhood quality and lower neighbourhood prosperity has also been shown to predict more mother reported mental-health problems [37]. With regard to social stressors at the household level, housing instability and disarray, rather than deterioration, has been shown to be associated with depression and generalised anxiety among women regardless of other social stressors present in their lives [13].

The strengths of the HICH study include: a) the measurement and assessment of the concurrent influence of a range of other related factors with the potential to confound or modify the association between housing condition and health outcomes; b) measurement of a range of important exposures as well as a range of outcomes; c) detailed assessment of the functional state of a wide range of items of housing infrastructure and of the hygienic condition of the household environment; and d) inclusion of multiple communities spread across a wide geographic area in order to enhance the potential generalisability of the findings, at least within the context of remote Australian Aboriginal communities [19]. The findings may be generalisable to some extent to indigenous communities in other developed countries, but generalisability to other settings is more questionable. The cohort analysis complements the cross sectional analysis of baseline data, providing additional explanatory rigor to the study. Limitations of the study include: a) the complexity of some of the constructs represented in the conceptual framework and the difficulty of defining appropriate and reliable measures for all constructs, specifically including the mental health of carers in the context of this study; b) the potential for respondent and recall bias associated with reliance on interview data for outcome and secondary exposure and confounding variables; c) the potential for chance associations in analyses involving large numbers of variables (although chance associations are limited by the use of hierarchical models and a focus on identifying consistent associations between exposure measures and a number of different health outcomes); d) the potential for measurement error/misclassification, particularly for some of the more complex constructs included in the analysis of explanatory factors; e) the potential for odds ratios to over-estimate the strength of associations for high prevalence exposures; and f) variation in the time between the surveys and the occupation of new houses, and variable and generally limited duration of follow-up. In addition, there was some loss to follow-up of carers who were included in the baseline survey, and we were not able to get complete data for all carers who were followed up (Figure 1). However, there was little difference between children in the cohort compared to those lost to follow-up across a range of variables [19] and data were missing for less than 8.5% of carers for all secondary explanatory variables.

## Conclusions

We have previously argued for the need for housing programs to be supported by social, behavioral and community-wide environmental interventions in order for the potential health gains of improved housing to be more fully realized [19]. The findings presented here add weight to that argument, and highlight the need for programs to reduce the incidence of negative life events for community residents – in general and particularly in relation to the occurrence of serious accidents, incidents that lead to incarceration of community residents, and in relation to community environmental conditions that contribute to the high mortality rates in these communities. There is a particular need for rigorously evaluated programs that aim to enhance social support for at risk groups and individuals (such as carers of young children), and to reduce the substantial and multiple sources of stress experienced by carers and families in these communities. There is also a need for large and longer-term studies involving multiple communities in a way that can effectively account for community or area level influences, that include qualitative, quantitative and participatory methods, that include other potentially important explanatory constructs such as mastery [38] and self-efficacy [39], and contribute to development of improved measures of community and self-reported health [40].

The study findings also have important implications for clinical practice, and highlight the importance of identifying carers who are at particularly high risk of poor mental health, including those who have experienced multiple negative life events; death, injury or incarceration of a family member or close friend; younger carers, those experiencing financial stress, lacking social support, and those with a child in day care. Clinicians need to be particularly alert to attending to the mental health needs of these carers – for the sake of the carers and their children. Efforts that are effective in improving the mental health of carers should have flow on effects for improving the health of children in these communities through a range of mechanisms [4].

## Competing interests

The authors declare that they have no competing interests.

## Authors’ contributions

RSB developed the study design and managed the implementation of the study. He developed the data analysis plan and played a primary role in the interpretation of study results and preparation of the manuscript. MS was responsible for data management and data analysis and contributed to preparation of the manuscript. ELM developed and implemented the pilot study on which this study is based. She provided expert advice and support in contextualising study findings and assisted with manuscript preparation. All authors read and approved the final manuscript.

## Pre-publication history

The pre-publication history for this paper can be accessed here:

http://www.biomedcentral.com/1471-2458/14/472/prepub

## Supplementary Material

Additional file 1 Unadjusted odds ratios (95% confidence interval) between socio-demographic variables and carer negative affect and risk of depression at baseline. Click here for file

Additional file 2 Unadjusted odds ratios (95% confidence interval) between child health and health behaviour and hygiene variables and carer negative affect and risk of depression at baseline. Click here for file

Additional file 3 Unadjusted odds ratios (95% confidence interval) between socioeconomic and financial stress variables and carer negative affect and risk of depression at baseline. Click here for file

Additional file 4 Unadjusted odds ratios (95% confidence interval) between psychosocial variables and carer negative affect and risk of depression at baseline. Click here for file

Additional file 5 Unadjusted linear regression between all significant explanatory variables and change (∆) in carer Negative affect and change in risk of depression between baseline and follow-up. Click here for file

## References

[B1] Surkan PJ Kennedy CE Hurley KM Black MM Maternal depression and early childhood growth in developing countries: systematic review and meta-analysis Bull World Health Organ 2011 89 608 615E 10.2471/BLT.11.088187 21836759 PMC3150769

[B2] Center on the Developing Child at Harvard University Maternal Depression Can Undermine the Development of Young Children: Working Paper No. 8 2009 Harvard: Center on the Developing Child at Harvard University 1 13

[B3] Stewart RC Maternal depression and infant growth: a review of recent evidence Matern Child Nutr 2007 3 2 94 107 10.1111/j.1740-8709.2007.00088.x 17355442 PMC6860855

[B4] Walker SP Wachs TD Gardner JM Lozoff B Wasserman GA Pollitt E Carter JA International Child Development Steering Group Child development: risk factors for adverse outcomes in developing countries Lancet 2007 369 9556 145 157 10.1016/S0140-6736(07)60076-2 17223478

[B5] Krieger J Higgins DL Housing and health: time again for public health action Am J Public Health 2002 92 5 758 768 10.2105/AJPH.92.5.758 11988443 PMC1447157

[B6] Lowry S An introduction to housing and health BMJ 1989 299 6710 1261 1262 10.1136/bmj.299.6710.1261 2513904 PMC1838118

[B7] U.S. Department of Health and Human Services The Surgeon General’s Call to Action To Promote Healthy Homes U.S. Department of Health and Human Services, Office of the Surgeon General 2009 1 66 20669408

[B8] Howden-Chapman PB Michael G Bierre S The houses children live in: policies to improve housing quality Pol Q 2013 9 9 35

[B9] Dickerson SS Kemeny ME Acute stressors and cortisol responses: a theoretical integration and synthesis of laboratory research Psychol Bull 2004 130 3 355 391 15122924 10.1037/0033-2909.130.3.355

[B10] Evans GW Wells NM Moch A Housing and mental health: a review of the evidence and a methodological and conceptual critique J Soc Issues 2003 59 3 475 500 10.1111/1540-4560.00074

[B11] Fuller TD Edwards JN Vorakitphokatorn S Sermsri S Chronic stress and psychological well-being: evidence from Thailand on household crowding Soc Sci Med 1996 42 2 265 280 10.1016/0277-9536(95)00089-5 8928035

[B12] Evans GW Behavioral and physiological consequences of crowding in humans1 J Appl Soc Psychol 1979 9 1 27 46 10.1111/j.1559-1816.1979.tb00793.x

[B13] Suglia SF Duarte CS Sandel MT Housing quality, housing instability, and maternal mental health J Urban Health 2011 88 6 1105 1116 10.1007/s11524-011-9587-0 21647798 PMC3232414

[B14] Thomson H Thomas S Sellstrom E Petticrew M Housing improvements for health and associated socio-economic outcomes Cochrane Collaboration 2013 2 1 189 10.1002/14651858.CD008657.pub2 PMC12551615 23450585

[B15] Thomson H Thomas S Sellstrom E Petticrew M Housing improvements for health and associated socio-economic outcomes: a systematic review Campbell Systematic Reviews 2013 2 348

[B16] Dunn JR Housing and health inequalities: review and prospects for research Hous Stud 2000 15 3 341 366 10.1080/02673030050009221

[B17] Bailie R Stevens M McDonald E Brewster D Guthridge S Exploring cross-sectional associations between common childhood illness, housing and social conditions in remote Australian Aboriginal communities BMC Public Health 2010 10 147 10.1186/1471-2458-10-147 20302661 PMC2848201

[B18] Bailie RS McDonald E Stevens M Guthridge S Brewster DR Evaluation of an Australian indigenous housing programme: community level impact on crowding, infrastructure function and hygiene J Epidemiol Community Health 2011 65 5 432 437 10.1136/jech.2009.091637 20466712 PMC3071088

[B19] Bailie RS Stevens M McDonald EL The impact of housing improvement and socio-environmental factors on common childhood illnesses: a cohort study in Indigenous Australian communities J Epidemiol Community Health 2012 66 9 821 831 10.1136/jech.2011.134874 21693472 PMC3412050

[B20] Kowal E Donohoe P Lonergan K Dust, distance and discussion: fieldwork experiences from the housing improvement and child health study Environ Health 2005 5 3 59 72

[B21] Kowal E Gunthorpe W Bailie RS Measuring emotional and social wellbeing in Aboriginal and Torres Strait Islander populations: an analysis of a negative life events scale Int J Equity Health 2007 6 18 10.1186/1475-9276-6-18 18001479 PMC2203968

[B22] Bailie RS Runcie MJ Household infrastructure in aboriginal communities and the implications for health improvement Med J Aust 2001 175 7 363 366 11700813 10.5694/j.1326-5377.2001.tb143619.x

[B23] Australian Institute of Health and Welfare and Australian Bureau of Statistics The Health and Welfare of Australia’s Aboriginal and Torres Strait Islander Peoples 2008 Canberra: Commonwealth of Australia 1 291

[B24] Australian Institute of Health and Welfare Child Protection Australia 2011–12. Cat. no. CWS 43 2013 Canberra: Commonwealth of Australia 1 169

[B25] Australian Institute of Health and Welfare The Health and Welfare of Australia’s Aboriginal and Torres Strait Islander People, An Overview 2011. Cat. no. IHW 42 2011 Canberra: Commonwealth of Australia 1 127

[B26] Bradburn NN Edward C The Structure of Psychological Well-being 1969 Chicago: Aldine 1 318

[B27] Hakstian AR McLean PD Brief screen for depression. Psychological assessment J Consult Clin Psychol 1989 1 2 139 141

[B28] Daniel M Cargo MD Lifshay J Green LW Cigarette smoking, mental health and social support: data from a northwestern first nation Can J Public Health 2004 95 1 45 49 14768741 10.1007/BF03403633 PMC6975667

[B29] Pholeros P Rianow S Torzillo P Housing for Health: Towards a Healthy Living Environment for Aboriginal Australia 1993 Newport Beach: Healthabitat Pty Ltd

[B30] Department of Families, Housing and Community services and Indigenous affairs, and Centre for Appropriate Technology National Indigenous Infrastructure Guide 2010 Canberra: Commonwealth of Australia

[B31] Australian Bureau of Statistics National Aboriginal and Torres Strait Islander Health Survey, 2004–2005. Cat. no. 4715.0 2006 Canberra: Commonwealth of Australia 1 78

[B32] Kelly K Dungeon P Gee G Glaskin B on behalf of the Australian Indigenous Psychologists Association Living on the Edge: Social and Emotional Wellbeing and Risk and Protective Factors for Serious Psychological Distress among Aboriginal and Torres Strait Islander People 2009 Discussion Paper No. 10, Cooperative Research Centre for Aboriginal Health, Darwin 1 42

[B33] Williams RL A note on robust variance estimation for cluster-correlated data Biometrics 2000 56 2 645 646 10.1111/j.0006-341X.2000.00645.x 10877330

[B34] Riva MN Gauvin L Barnett T Toward the next generation of research into small area effects on health: a synthesis of multilevel investigations published since July 1998 J Epidemiol Community Health 2007 61 10 853 861 10.1136/jech.2006.050740 17873220 PMC2652961

[B35] Mair C Diez Roux AV Galea S Are neighbourhood characteristics associated with depressive symptoms? a review of evidence J Epidemiol Community Health 2008 62 11 940 946 8 p following 946 18775943 10.1136/jech.2007.066605

[B36] Kohen DE Leventhal T Dahinten VS McIntosh CN Neighborhood disadvantage: pathways of effects for young children Child Dev 2008 79 1 156 169 10.1111/j.1467-8624.2007.01117.x 18269515

[B37] Barnes J Belsky J Frost M Melhuish E Neighborhood characteristics and mental health: the relevance for mothers of infants in deprived English neighborhoods Soc Psychiatry Psychiatr Epidemiol 2011 46 12 1243 1249 10.1007/s00127-010-0298-8 20924554

[B38] Scholz U Guitierrez Dona B Sud S Schwarzer R Is general self-efficacy and universal construct? Eur J Psychol Assess 2002 18 3 242 251 10.1027//1015-5759.18.3.242

[B39] Pearlin LI Lieberman MA Menaghan EG Mullan JT The stress process J Health Soc Behav 1981 22 337 356 10.2307/2136676 7320473

[B40] Sibthorpe B Anderson I Cunningham J Self-assessed health among indigenous Australians: how valid is a global question? Am J Public Health 2001 91 10 1660 1663 10.2105/AJPH.91.10.1660 11574332 PMC1446851

