# Supplementary material for: Impact of housing improvement and the socio-physical environment on the mental health of children’s carers: a cohort study in Australian Aboriginal communities
Source: BMC Public Health. 2014 May 19;14:472. doi: 10.1186/1471-2458-14-472 (PMC4060879; doi:10.1186/1471-2458-14-472)
Supplement: Additional file 1 — Unadjusted odds ratios (95% confidence interval) between socio-demographic variables and carer negative affect and risk of depression at baseline. [file 1471-2458-14-472-S1.doc]

**Additional File 1**. Unadjusted odds ratios (95% confidence interval) between socio-demographic variables and carer negative affect and risk of depression at baseline

|  |  |  |  | **High negative affect** | |  | **High risk of depression** | |
| --- | --- | --- | --- | --- | --- | --- | --- | --- |
| **Carer socio-demographic** | **Missing**  **n (%)** | **Carers**  **n (%)** |  | **n (%)^1^** | **OR (95% CI)** |  | **n (%)^1^** | **OR (95% CI)** |
| **All carers** |  | **328 (100)** |  | **75 (22.9)** |  |  | **53 (16.2)** |  |
| Community ID |  |  |  |  |  |  |  |  |
| 1 | 0 (0.0) | 48 (14.6) |  | 6 (12.5) | 1.0 |  | 9 (18.8) | 1.0 |
| 2 |  | 34 (10.4) |  | 6 (17.7) | 1.50 (0.44-5.13) |  | 5 (14.7) | 0.75 (0.22-2.54) |
| 3 |  | 32 (9.8) |  | 17 (53.1) | **7.93 (2.85-22.1)** |  | 7 (21.9) | 1.21 (0.37-3.95) |
| 4 |  | 19 (5.8) |  | 4 (21.1) | 1.87 (0.56-6.23) |  | 1 (5.3) | 0.24 (0.03-2.14) |
| 5 |  | 44 (13.4) |  | 15 (34.1) | **3.62 (1.35-9.75)** |  | 9 (20.5) | 1.11 (0.40-3.08) |
| 6 |  | 9 (2.7) |  | 1 (11.1) | 0.88 (0.09-8.28) |  | 1 (11.1) | 0.54 (0.06-4.97) |
| 7 |  | 50 (15.2) |  | 5 (10.0) | 0.78 (0.22-2.75) |  | 9 (18.0) | 0.95 (0.32-2.84) |
| 8 |  | 57 (17.4) |  | 13 (22.8) | 2.07 (0.76-5.66) |  | 4 (7.0) | 0.33 (0.09-1.13) |
| 9 |  | 22 (6.7) |  | 6 (27.3) | 2.63 (0.72-9.51) |  | 3 (13.6) | 0.68 (0.16-2.92) |
| 10 |  | 13 (4.0) |  | 2 (15.4) | 1.27 (0.25-6.46) |  | 5 (38.5) | 2.71 (0.63-11.7) |
| *Carer socio-demographic* |  |  |  |  |  |  |  |  |
| Carer sex |  |  |  |  |  |  |  |  |
| Male | 0 (0.0) | 17 (5.2) |  | 5 (29.4) | 1.0 |  | 1 (5.9) | 1.0 |
| Female |  | 311 (94.8) |  | 70 (22.5) | 0.70 (0.23-2.10) |  | 52 (16.7) | 3.21 (0.41-25.1) |
| Carer age |  |  |  |  |  |  |  |  |
| 20-34 years | 27 (8.2) | 197 (65.4) |  | 47 (23.9) | 1.0 |  | 29 (14.7) | 1.0 |
| Less than 20 years |  | 27 (9.0) |  | 5 (18.5) | 0.73 (0.26-2.04) |  | 4 (14.8) | 1.01 (0.32-3.14) |
| 35+ years |  | 77 (25.6) |  | 16 (20.8) | 0.84 (0.44-1.59) |  | 15 (19.5) | 1.40 (0.73-2.70) |
| Carer spousal cohabitation |  |  |  |  |  |  |  |  |
| No | 0 (0.0) | 80 (24.4) |  | 19 (23.8) | 1.0 |  | 15 (18.8) | 1.0 |
| Yes – living with spouse |  | 187 (57.0) |  | 46 (24.6) | 1.05 (0.57-1.92) |  | 29 (15.5) | 0.80 (0.41-1.54) |
| Don’t know/not sure |  | 61 (18.6) |  | 10 (16.4) | 0.63 (0.27-1.47) |  | 9 (14.8) | 0.75 (0.29-1.92) |
| Carer relationship to householder |  |  |  |  |  |  |  |  |
| Carer is main householder | 6 (1.8) | 72 (22.4) |  | 13 (18.1) | 1.0 |  | 15 (20.8) | (p=0.063) 1.0 |
| Daughter |  | 85 (26.4) |  | 20 (23.5) | 1.40 (0.66-2.96) |  | 7 (8.2) | **0.34 (0.13-0.87)** |
| Wife |  | 63 (19.6) |  | 13 (20.6) | 1.18 (0.50-2.78) |  | 8 (12.7) | 0.55 (0.22-1.41) |
| Daughter-in-law |  | 20 (6.2) |  | 4 (20.0) | 1.13 (0.34-3.76) |  | 4 (20.0) | 0.95 (0.27-3.30) |
| Niece |  | 20 (6.2) |  | 6 (30.0) | 1.95 (0.64-5.89) |  | 3 (15.0) | 0.67 (0.18-2.48) |
| Sister |  | 15 (4.7) |  | 2 (13.3) | 0.70 (0.14-3.53) |  | 2 (13.3) | 0.58 (0.12-2.92) |
| Sister-in-law |  | 10 (3.1) |  | 2 (20.0) | 1.13 (0.21-6.12) |  | 3 (30.0) | 1.63 (0.38-7.04) |
| Other |  | 37 (11.5) |  | 12 (32.4) | 2.18 (0.88-5.37) |  | 10 (27.0) | 1.41 (0.56-3.57) |
| Carer lived in house for more than one year |  |  |  |  |  |  |  |  |
| No | 0 (0.0) | 61 (18.6) |  | 21 (34.4) | 1.0 |  | 10 (16.4) | 1.0 |
| Yes |  | 267 (81.4) |  | 54 (20.2) | **0.48 (0.25-0.93)** |  | 43 (16.1) | 0.98 (0.46-2.07) |
| Lived in other community for more than 4 weeks |  |  |  |  |  |  |  |  |
| No | 0 (0.0) | 249 (75.9) |  | 51 (20.5) | 1.0 |  | 40 (16.1) | 1.0 |
| Yes |  | 79 (24.1) |  | 24 (30.4) | 1.69 (0.95-3.04) |  | 13 (16.5) | 1.03 (0.53-2.02) |
| Number of children cared for less than one year |  |  |  |  |  |  |  |  |
| None | 0 (0.0) | 245 (74.7) |  | 63 (25.7) | 1.0 |  | 41 (16.7) | 1.0 |
| One to four |  | 83 (25.3) |  | 12 (14.5) | **0.49 (0.25-0.94)** |  | 12 (14.5) | 0.84 (0.42-1.67) |
| Number of children cared for aged 1-3 years |  |  |  |  |  |  |  |  |
| None | 0 (0.0) | 118 (36) |  | 18 (15.3) | 1.0 |  | 17 (14.4) | 1.0 |
| One to three |  | 210 (64) |  | 57 (27.1) | **2.07 (1.17-3.66)** |  | 36 (17.1) | 1.23 (0.67-2.24) |
| Number of children cared for aged 4-7 years |  |  |  |  |  |  |  |  |
| None | 0 (0.0) | 139 (42.4) |  | 29 (20.9) | 1.0 |  | 22 (15.8) | 1.0 |
| One to three |  | 189 (57.6) |  | 46 (24.3) | 1.22 (0.73-2.05) |  | 31 (16.4) | 1.04 (0.56-1.93) |
| Number of children cared for aged 8 to 15 years |  |  |  |  |  |  |  |  |
| None | 7 (2.1) | 163 (50.8) |  | 48 (29.5) | 1.0 |  | 24 (14.7) | 1.0 |
| One |  | 62 (19.3) |  | 12 (19.4) | 0.58 (0.28-1.18) |  | 13 (21.0) | 1.54 (0.73-3.25) |
| Two to nine |  | 96 (29.9) |  | 14 (14.6) | **0.41 (0.21-0.78)** |  | 15 (15.6) | 1.07 (0.51-2.27) |
| Number of adults in house^2^ |  |  |  |  |  |  |  |  |
| 2-4 | 13 (4.0) | 103 (32.7) |  | 26 (25.2) | 1.0 |  | 15 (14.6) | 1.0 |
| 5-6 |  | 99 (31.4) |  | 24 (24.2) | 0.95 (0.51-1.77) |  | 17 (17.2) | 1.22 (0.55-2.70) |
| 7-15 |  | 113 (35.9) |  | 22 (19.5) | 0.72 (0.40-1.30) |  | 20 (17.7) | 1.26 (0.61-2.63) |

^1^ Number and percentage of carers classified as having high negative affect or being at high risk of depression

^2^ No houses had less than 2 adults

Bold font indicates the variable was significant at p0.05
