# Supplementary material for: Impact of housing improvement and the socio-physical environment on the mental health of children’s carers: a cohort study in Australian Aboriginal communities
Source: BMC Public Health. 2014 May 19;14:472. doi: 10.1186/1471-2458-14-472 (PMC4060879; doi:10.1186/1471-2458-14-472)
Supplement: Additional file 2 — Unadjusted odds ratios (95% confidence interval) between child health and health behaviour and hygiene variables and carer negative affect and risk of depression at baseline. [file 1471-2458-14-472-S2.doc]

**Additional File 2**. Unadjusted odds ratios (95% confidence interval) between child health and health behaviour and hygiene variables and carer negative affect and risk of depression at baseline

|  |  |  |  | **High negative affect** | |  | **High risk of depression** | |
| --- | --- | --- | --- | --- | --- | --- | --- | --- |
|  | **Missing**  **n (%)** | **Carers**  **n (%)** |  | **n (%)** | **OR (95% CI)** |  | **n (%)** | **OR (95% CI)** |
| **All carers** | **0 (0.0)** | **328 (100)** |  | **75 (22.9)** | - |  | **53 (16.2)** | **-** |
| *Carer report of illness in previous 2 weeks* |  |  |  |  |  |  |  |  |
| Number of illnesses in carer’s children |  |  |  |  |  |  |  |  |
| None | 0 (0.0) | 83 (25.3) |  | 12 (14.5) | 1.0 |  | 11 (13.3) | 1.0 |
| 1 to 2 |  | 133 (40.5) |  | 30 (22.6) | 1.72 (0.82-3.64) |  | 18 (13.5) | 1.02 (0.46-2.30) |
| 3 to 4 |  | 71 (21.6) |  | 18 (25.4) | 2.01 (0.87-4.66) |  | 11 (15.5) | 1.20 (0.48-3.00) |
| 5 to 15 |  | 41 (12.5) |  | 15 (36.6) | **3.41 (1.36-8.55)** |  | 13 (31.7) | **3.04 (1.20-7.70)** |
| **Health behaviour, hygiene and day care** |  |  |  |  |  |  |  |  |
| Broom, mop and bucket |  |  |  |  |  |  |  |  |
| Absent | 37 (11.3) | 108 (37.1) |  | 26 (24.1) | 1.0 |  | 13 (11.9) | 1.0 |
| Present |  | 183 (62.9) |  | 42 (23.0) | 0.94 (0.56-1.58) |  | 36 (19.8) | 1.82 (0.92-3.60) |
| Soap |  |  |  |  |  |  |  |  |
| Absent | 23 (7.0) | 128 (42.0) |  | 26 (20.3) | 1.0 |  | 23 (18.0) | 1.0 |
| Present |  | 177 (58.0) |  | 42 (23.7) | 1.22 (0.73-2.04) |  | 27 (15.3) | 0.82 (0.44-1.55) |
| Overall Surveyor Condition Score^2^ |  |  |  |  |  |  |  |  |
| Better (scores 1-2) | 16 (4.9) | 50 (16.0) |  | 8 (16.0) | 1.0 |  | 7 (14.3) | 1.0 |
| Worst (scores 3-7) |  | 262 (84.0) |  | 61 (23.3) | 1.59 (0.74-3.44) |  | 44 (16.7) | 1.21 (0.51-2.87) |
| Number of children in day-care |  |  |  |  |  |  |  |  |
| None | 0 (0.0) | 284 (86.6) |  | 60 (21.1) | 1.0 |  | 44 (15.5) | 1.0 |
| One to three |  | 44 (13.4) |  | 15 (34.1) | 1.93 (0.96-3.89) |  | 9 (20.5) | 1.40 (0.62-3.20) |

^1^ Number and percentage of carers classified as having high negative affect or being at high risk of depression

^2^ Hygienic state of infrastructure components required to conduct Healthy Living Practices (HLPs) were observed by the surveyor

Bold font indicates the variable was significant at p0.05
