# Supplementary material for: Impact of housing improvement and the socio-physical environment on the mental health of children’s carers: a cohort study in Australian Aboriginal communities
Source: BMC Public Health. 2014 May 19;14:472. doi: 10.1186/1471-2458-14-472 (PMC4060879; doi:10.1186/1471-2458-14-472)
Supplement: Additional file 3 — Unadjusted odds ratios (95% confidence interval) between socioeconomic and financial stress variables and carer negative affect and risk of depression at baseline. [file 1471-2458-14-472-S3.doc]

**Additional File 3**. Unadjusted odds ratios (95% confidence interval) between socioeconomic and financial stress variables and carer negative affect and risk of depression at baseline

|  |  |  |  | **High negative affect** | |  | **High risk of depression** | |
| --- | --- | --- | --- | --- | --- | --- | --- | --- |
| **Carer socioeconomic status**  **and financial stress** | **Missing**  **n (%)** | **Carers**  **n (%)** |  | **n (%)^1^** | **OR (95% CI)** |  | **n (%)^1^** | **OR (95% CI)** |
| **All carers** | **0 (0.0)** | **328 (100)** |  | **75 (22.9)** | - |  | **53 (16.2)** | **-** |
| Carer highest schooling |  |  |  |  |  |  |  |  |
| Years 11 and 12 | 0 (0.0) | 117 (35.7) |  | 31 (26.5) | 1.0 |  | 17 (14.5) | 1.0 |
| Years 9 and 10 |  | 171 (52.1) |  | 35 (20.5) | 0.71 (0.41-1.23) |  | 31 (18.1) | 1.30 (0.68-2.49) |
| Year 8 or less |  | 40 (12.2) |  | 9 (22.5) | 0.81 (0.34-1.91) |  | 5 (12.5) | 0.84 (0.29-2.47) |
| Carer labour force status |  |  |  |  |  |  |  |  |
| Employed | 1 (0.3) | 22 (6.7) |  | 8 (36.4) | 1.0 |  | 4 (18.2) | 1.0 |
| CDEP |  | 58 (17.7) |  | 16 (27.6) | 0.67 (0.23-1.91) |  | 10 (17.2) | 0.94 (0.25-3.45) |
| Unemployed |  | 132 (40.4) |  | 29 (22.0) | 0.49 (0.19-1.30) |  | 21 (15.9) | 0.85 (0.26-2.82) |
| Pension |  | 7 (2.1) |  | 1 (14.3) | 0.29 (0.03-2.89) |  | 4 (57.1) | 6.00 (0.94-38.2) |
| Parenting payment/Family Allowance |  | 108 (33.0) |  | 21 (19.4) | 0.42 (0.16-1.14) |  | 14 (13.0) | 0.67 (0.19-2.31) |
| Household material wealth |  |  |  |  |  |  |  |  |
| Neither phone or fridge | 16 (4.9) | 89 (28.5) |  | 18 (20.2) | 1.0 |  | 12 (13.5) | 1.0 |
| Either phone or fridge |  | 169 (54.2) |  | 39 (23.1) | 1.18 (0.65-2.14) |  | 34 (20.0) | 1.60 (0.79-3.26) |
| Has phone and fridge |  | 54 (17.3) |  | 12 (22.2) | 1.13 (0.50-2.52) |  | 5 (9.4) | 0.67 (0.23-1.95) |
| Householder holds important position |  |  |  |  |  |  |  |  |
| None | 10 (3.0) | 137 (43.1) |  | 29 (21.2) | 1.0 |  | 27 (19.7) | 1.0 |
| One |  | 76 (23.9) |  | 21 (27.6) | 1.42 (0.76-2.65) |  | 15 (19.7) | 1.00 (0.48-2.10) |
| 2 to 3 |  | 57 (17.9) |  | 12 (21.1) | 0.99 (0.48-2.05) |  | 5 (8.8) | 0.39 (0.14-1.08) |
| 4 to 8 |  | 48 (15.1) |  | 10 (20.8) | 0.98 (0.48-2.00) |  | 5 (10.4) | 0.47 (0.18-1.26) |
| Ran out of money in last 2 weeks |  |  |  |  |  |  |  |  |
| No | 1 (0.3) | 176 (53.8) |  | 32 (18.2) | 1.0 |  | 23 (13.1) | 1.0 |
| Yes |  | 151 (46.2) |  | 43 (28.5) | **1.79 (1.08-2.97)** |  | 30 (19.9) | 1.65 (0.91-2.97) |
| Ran out of money in last year |  |  |  |  |  |  |  |  |
| No | 2 (0.6) | 102 (31.3) |  | 22 (21.6) | 1.0 |  | 11 (10.8) | 1.0 |
| Yes |  | 224 (68.7) |  | 53 (23.7) | 1.13 (0.65-1.97) |  | 42 (18.8) | 1.91 (0.98-3.72) |
| Number of things did to get money if ran out |  |  |  |  |  |  |  |  |
| None – had money | 4 (1.2) | 102 (31.5) |  | 22 (21.6) | 1.0 |  | 11 (10.8) | (p=0.069) 1.0 |
| None |  | 6 (1.9) |  | 0 (0.0) | **ne** |  | 0 (0.0) | **ne** |
| 1 or 2 things |  | 109 (33.6) |  | 23 (21.1) | 0.97 (0.51-1.86) |  | 18 (16.5) | 1.64 (0.76-3.54) |
| Three or more |  | 107 (33.0) |  | 30 (28.0) | 1.42 (0.76-2.64) |  | 24 (22.4) | **2.39 (1.14-5.01)** |
| Raise $2000 in a week for emergency |  |  |  |  |  |  |  |  |
| Non | 2 (0.6) | 224 (68.7) |  | 50 (22.3) | 1.0 |  | 35 (15.6) | 1.0 |
| Yes |  | 72 (22.1) |  | 18 (25.0) | 1.16 (0.62-2.16) |  | 14 (19.4) | 1.30 (0.66-2.57) |
| Don’t know |  | 30 (9.2) |  | 7 (23.3) | 1.06 (0.43-2.63) |  | 4 (13.3) | 0.83 (0.28-2.48) |

^1^ Number and percentage of carers classified as having high negative affect or being at high risk of depression

Bold font indicates the variable was significant at p0.05; ne – not able to be estimated
