# Supplementary material for: Impact of housing improvement and the socio-physical environment on the mental health of children’s carers: a cohort study in Australian Aboriginal communities
Source: BMC Public Health. 2014 May 19;14:472. doi: 10.1186/1471-2458-14-472 (PMC4060879; doi:10.1186/1471-2458-14-472)
Supplement: Additional file 4 — Unadjusted odds ratios (95% confidence interval) between psychosocial variables and carer negative affect and risk of depression at baseline. [file 1471-2458-14-472-S4.doc]

**Additional File 4**. Unadjusted odds ratios (95% confidence interval) between psychosocial variables and carer negative affect and risk of depression at baseline

|  |  |  |  | **High negative affect** | |  | **High risk of depression** | |
| --- | --- | --- | --- | --- | --- | --- | --- | --- |
| **Psychosocial and health** | **Missing**  **n (%)** | **Carers**  **n (%)** |  | **n (%)^1^** | **OR (95% CI)** |  | **n (%)^1^** | **OR (95% CI)** |
| **All carers** | **0 (0.0)** | **328 (100)** |  | **75 (22.9)** | - |  | **53 (16.2)** | **-** |
| Other people from tribal group live in community |  |  |  |  |  |  |  |  |
| No | 3 (0.9) | 26 (8.0) |  | 3 (11.5) | 1.0 |  | 3 (11.5) | 1.0 |
| Yes |  | 299 (92.0) |  | 72 (24.1) | 2.43 (0.70-8.43) |  | 49 (16.4) | 1.50 (0.43-5.24) |
| Frequency of visits to traditional land |  |  |  |  |  |  |  |  |
| Lives on | 7 (2.1) | 129 (40.2) |  | 32 (24.8) | 1.0 |  | 23 (17.8) | 1.0 |
| 5 or more per year |  | 39 (12.1) |  | 6 (15.4) | 0.55 (0.23-1.35) |  | 8 (20.5) | 1.19 (0.51-2.80) |
| 2 to 4 per year |  | 26 (8.1) |  | 4 (15.4) | 0.55 (0.17-1.79) |  | 1 (3.9) | 0.18 (0.02-1.46) |
| Less than equal to 1 per year |  | 127 (39.6) |  | 32 (25.2) | 1.02 (0.58-1.80) |  | 19 (15.0) | 0.81 (0.42-1.58) |
| Number of people get help from if has serious worries |  |  |  |  |  |  |  |  |
| None | 0 (0.0) | 113 (34.5) |  | 17 (15.0) | 1.0 |  | 21 (18.6) | 1.0 |
| One |  | 89 (27.1) |  | 18 (20.2) | 1.43 (0.68-3.00) |  | 11 (12.4) | 0.62 (0.26-1.44) |
| Two or more |  | 126 (38.4) |  | 40 (31.8) | **2.63 (1.39-4.98)** |  | 21 (16.7) | 0.88 (0.44-1.74) |
| Carer self-reported health |  |  |  |  |  |  |  |  |
| Excellent/very good | 4 (1.2) | 120 (37.0) |  | 32 (26.7) | 1.0 |  | 15 (12.5) | 1.0 |
| Good |  | 147 (45.4) |  | 34 (23.1) | 0.83 (0.47-1.45) |  | 22 (15.0) | 1.23 (0.61-2.51) |
| Okay/bad |  | 57 (17.6) |  | 9 (15.8) | 0.52 (0.23-1.15) |  | 13 (22.8) | 2.07 (0.91-4.71) |
| *Carer NLES items* |  |  |  |  |  |  |  |  |
| Worried about someone sick/disabled |  |  |  |  |  |  |  |  |
| No | 2 (0.6) | 156 (47.9) |  | 32 (20.5) | 1.0 |  | 24 (15.4) | 1.0 |
| Yes |  | 170 (52.1) |  | 43 (25.3) | 1.31 (0.79-2.17) |  | 29 (17.1) | 1.13 (0.61-2.09) |
| Know someone who had a bad accident |  |  |  |  |  |  |  |  |
| No | 2 (0.6) | 228 (69.9) |  | 38 (16.7) | 1.0 |  | 30 (13.2) | 1.0 |
| Yes |  | 98 (30.1) |  | 37 (37.8) | **3.03 (1.77-5.19)** |  | 23 (23.5) | **2.02 (1.08-3.80)** |
| Death of family member or close friend |  |  |  |  |  |  |  |  |
| No | 2 (0.6) | 98 (30.1) |  | 13 (13.3) | 1.0 |  | 7 (7.1) | 1.0 |
| Yes |  | 228 (69.9) |  | 62 (27.2) | **2.44 (1.26-4.73)** |  | 46 (20.2) | **3.29 (1.43-7.54)** |
| Member of family in jail or sent to jail |  |  |  |  |  |  |  |  |
| No | 2 (0.6) | 215 (66.0) |  | 42 (19.5) | 1.0 |  | 31 (14.4) | 1.0 |
| Yes |  | 111 (34.0) |  | 33 (29.7) | **1.74 (1.03-2.96)** |  | 22 (19.8) | 1.47 (0.77-2.79) |
| Too many people living in one house |  |  |  |  |  |  |  |  |
| No | 2 (0.6) | 87 (26.7) |  | 20 (23.0) | 1.0 |  | 12 (13.8) | 1.0 |
| Yes |  | 239 (73.3) |  | 55 (23.0) | 1.00 (0.56-1.81) |  | 41 (17.2) | 1.29 (0.64-2.62) |
| Worried about divorce/separation |  |  |  |  |  |  |  |  |
| No | 2 (0.6) | 274 (84.0) |  | 62 (22.6) | 1.0 |  | 41 (15.0) | 1.0 |
| Yes |  | 52 (16.0) |  | 13 (25.0) | 1.14 (0.58-2.23) |  | 12 (23.1) | 1.70 (0.76-3.80) |
| Not able to get a job |  |  |  |  |  |  |  |  |
| No | 2 (0.6) | 283 (86.8) |  | 61 (21.6) | 1.0 |  | 45 (15.9) | 1.0 |
| Yes |  | 43 (13.2) |  | 14 (32.6) | 1.76 (0.87-3.54) |  | 8 (18.6) | 1.21 (0.53-2.78) |
| Lost their job/sacked |  |  |  |  |  |  |  |  |
| No | 2 (0.6) | 314 (96.3) |  | 67 (21.3) | 1.0 |  | 48 (15.3) | 1.0 |
| Yes |  | 12 (3.7) |  | 8 (66.7) | **7.37 (2.14-25.4)** |  | 5 (41.7) | **3.96 (1.21-13.0)** |
| Alcohol or drug problems |  |  |  |  |  |  |  |  |
| No | 2 (0.6) | 176 (54) |  | 30 (17.1) | 1.0 |  | 19 (10.8) | 1.0 |
| Yes |  | 150 (46) |  | 45 (30.0) | **2.09 (1.23-3.53)** |  | 34 (22.7) | **2.42 (1.27-4.62)** |
| Seeing fights and people beaten up |  |  |  |  |  |  |  |  |
| No | 2 (0.6) | 101 (31.0) |  | 16 (15.8) | 1.0 |  | 12 (11.9) | 1.0 |
| Yes |  | 225 (69.0) |  | 59 (26.2) | **1.89 (1.04-3.44)** |  | 41 (18.2) | 1.65 (0.82-3.34) |
| Someone being abused or victim of violent crime |  |  |  |  |  |  |  |  |
| No | 3 (0.9) | 174 (53.5) |  | 31 (17.8) | 1.0 |  | 20 (11.5) | 1.0 |
| Yes |  | 151 (46.5) |  | 43 (28.5) | **1.84 (1.09-3.10)** |  | 33 (21.9) | **2.15 (1.18-3.94)** |
| Trouble with police |  |  |  |  |  |  |  |  |
| No | 3 (0.9) | 251 (77.2) |  | 45 (17.9) | 1.0 |  | 39 (15.5) | 1.0 |
| Yes |  | 74 (22.8) |  | 30 (40.5) | **3.12 (1.75-5.57)** |  | 14 (18.9) | 1.27 (0.64-2.52) |
| Gambling problems |  |  |  |  |  |  |  |  |
| No | 3 (0.9) | 189 (58.2) |  | 25 (13.2) | 1.0 |  | 25 (13.2) | 1.0 |
| Yes |  | 136 (41.8) |  | 50 (36.8) | **3.81 (2.21-6.58)** |  | 28 (20.6) | 1.70 (0.90-3.20) |
| Racism |  |  |  |  |  |  |  |  |
| No | 4 (1.2) | 214 (66.0) |  | 36 (16.8) | 1.0 |  | 33 (15.4) | 1.0 |
| Yes |  | 110 (34.0) |  | 39 (35.5) | **2.72 (1.60-4.62)** |  | 20 (18.2) | 1.22 (0.67-2.20) |
| Carer NLES quartiles (number of stressors reported) |  |  |  |  |  |  |  |  |
| 0-3 | 5 (1.5) | 84 (26.0) |  | 9 (10.7) | 1.0 |  | 5 (6.0) | 1.0 |
| 4-5 |  | 74 (22.9) |  | 11 (14.9) | 1.46 (0.56-3.81) |  | 11 (14.9) | 2.76 (0.90-8.42) |
| 6-8 |  | 88 (27.2) |  | 23 (26.1) | **2.95 (1.24-6.99)** |  | 19 (21.6) | **4.35 (1.51-12.5)** |
| 9-13 |  | 77 (23.8) |  | 31 (40.3) | **5.62 (2.45-12.9)** |  | 18 (23.4) | **4.82 (1.64-14.2)** |

^1^ Number and percentage of carers classified as having high negative affect or being at high risk of depression

Bold font indicates the variable was significant at p0.05
