# Supplementary material for: Impact of housing improvement and the socio-physical environment on the mental health of children’s carers: a cohort study in Australian Aboriginal communities
Source: BMC Public Health. 2014 May 19;14:472. doi: 10.1186/1471-2458-14-472 (PMC4060879; doi:10.1186/1471-2458-14-472)
Supplement: Additional file 5 — Unadjusted linear regression between all significant explanatory variables and change (∆) in carer Negative affect and change in risk of depression between baseline and follow-up. [file 1471-2458-14-472-S5.doc]

**Additional File 5:** Unadjusted linear regression between all significant explanatory variables and change () in carer Negative affect and change in risk of depression between baseline and follow-up

| **Domain: Variable** | **Status at baseline or change**  **between baseline and follow up** | **Missing**  **n (%)** | **Carers**  **n (%)** | **Negative Affect**  **(95% CI)** | **Missing**  **n (%)** | **Carers**  **n (%)** | **Risk of depression**  **(95% CI)** |
| --- | --- | --- | --- | --- | --- | --- | --- |
| **All carers** | **Category** | **42 (20.2)** | **166 (100.0)** | **Mean = -0.05** | **57 (27.4)** | **151 (100.0)** | **Mean = -2.47** |
| **Primary explanatory variables** |  |  |  |  |  |  |  |
| Change in overall HLP measure | Limited or no change | 55 (33.1) | 61 (55.0) | 0.0 | 45 (29.8) | 57 (53.8) | 0.0 |
|  | Deterioration of 2 or more |  | 13 (11.7) | 0.09 (-0.81, 0.99) |  | 13 (12.3) | -1.84 (-6.72, 3.05) |
|  | Improvement of 2 or more |  | 37 (33.3) | **0.80 (0.12, 1.48)** |  | 36 (34.0) | 1.09 (-3.31, 5.49) |
| **Socio-demographic variables** |  |  |  |  |  |  |  |
| Community ID | 1 | 0 (0.0) | 21 (12.7) | -0.30 (-1.15, 0.54) | 0 (0.0) | 21 (13.9) | 0.72 (-3.30, 4.74) |
|  | 2 |  | 20 (12.1) | **-0.88 (-1.73, -0.02)** |  | 21 (13.9) | 3.01 (-2.37, 8.39) |
|  | 3 |  | 25 (15.1) | 0.25 (-0.45, 0.94) |  | 27 (17.9) | **6.79 (1.77, 11.82)** |
|  | 4 |  | 9 (5.4) | 1.13 (-0.08, 2.33) |  | 9 (6.0) | 3.64 (-3.43, 10.72) |
|  | 5 |  | 17 (10.2) | **2.01 (1.23, 2.78)** |  | 14 (9.3) | **7.84 (2.34, 13.35)** |
|  | 6 |  | 6 (3.6) | -0.21 (-1.29, 0.88) |  | 6 (4.0) | **4.53 (0.32, 8.74)** |
|  | 7 |  | 27 (16.3) | **-0.84 (-1.65, -0.02)** |  | 25 (16.6) | **4.96 (1.30, 8.62)** |
|  | 8 (NegAB = 0.13, BSD = 6.2) |  | 32 (19.3) | 0.0 |  | 20 (13.3) | 0.0 |
|  | 9 |  | 5 (3.0) | 0.33 (-1.35, 2.00) |  | 5 (3.3) | 2.60 (-6.27, 11.47) |
|  | 10 |  | 4 (2.4) | **1.88 (1.15, 2.60)** |  | 3 (2.0) | -1.80 (-7.31, 3.71) |
| Carer: age at baseline | 20-34yrs | 8 (4.8) | 110 (69.6) | 0.0 | 8 (5.3) | 98 (68.5) | 0.0 |
|  | <20yrs |  | 9 (5.7) | -0.74 (-1.52, 0.04) |  | 7 (4.9) | **-8.23 (-14.77, -1.70)** |
|  | 35+yrs |  | 39 (24.7) | 0.04 (-0.65, 0.72) |  | 38 (26.6) | 1.03 (-2.52, 4.58) |
| Change in child day care attendance (children <6 at follow up) | No change | 0 (0.0) | 123 (74.1) | 0.0 | 0 (0.0) | 116 (76.8) | 0.0 |
|  | Change from No to Yes |  | 33 (19.9) | 0.28 (-0.39, 0.95) |  | 27 (17.9) | **-4.79 (-7.45, -2.12)** |
|  | Change from Yes to No |  | 10 (6.0) | **1.29 (0.52, 2.06)** |  | 8 (5.3) | -2.65 (-7.94, 2.64) |
| Carer: Change in householder | No change | 0 (0.0) | 120 (72.3) | 0.0 | 0 (0.0) | 108 (71.5) | 0.0 |
|  | Householder changed |  | 46 (27.7) | **0.74 (0.18, 1.29)** |  | 43 (28.5) | 1.47 (-2.30, 5.24) |
| Change in carer lives with spouse | Lives with spouse time 1 and 2 | 0 (0.0) | 93 (56.0) | 0.0 | 0 (0.0) | 89 (58.9) | 0.0 |
|  | Didn’t live with spouse to did live |  | 27 (16.3) | **-1.02 (-1.72, -0.32)** |  | 25 (16.6) | -3.30 (-7.51, 0.91) |
|  | Other |  | 46 (27.7) | -0.33 (-0.91, 0.24) |  | 37 (24.5) | -0.74 (-4.97, 3.48) |
| **Socioeconomic status and financial stress** |  |  |  |  |  |  |  |
| Change in number of important positions householder holds | Other | 25 (15.1) | 126 (89.4) | 0.0 | 21 (13.9) | 114 (87.7) | 0.0 |
|  | From 0/1 position to 2 or more positions |  | 15 (10.6) | **-0.92 (-1.82, -0.01)** |  | 16 (12.3) | -2.29 (-5.63, 1.05) |
| Change in carer ran out of money last year | Other | 2 (1.2) | 136 (82.9) | 0.0 | 2 (1.3) | 120 (80.5) | 0.0 |
|  | Change to ran out of money last year |  | 28 (17.1) | **-0.90 (-1.50, -0.29)** |  | 29 (19.5) | -3.79 (-8.21, 0.63) |
| Change in number of things did to get money | Other | 9 (5.4) | 130 (82.8) | 0.0 | 8 (5.3) | 116 (81.1) | 0.0 |
|  | Change to doing 3 or more things |  | 27 (17.2) | **-0.90 (-1.49, -0.31)** |  | 27 (18.9) | 3.29 (-1.00, 7.58) |
| **Psychosocial and health** |  |  |  |  |  |  |  |
| Change in carer’s has clan/kin in community | Other | 4 (2.4) | 153 (94.4) | 1.0 | 4 (2.7) | 137 (93.2) | 0.0 |
|  | Had clan/kin in community to did not |  | 9 (5.6) | -0.04 (-0.99, 0.91) |  | 10 (6.8) | **-4.85 (-8.25, -1.45)** |
| Change in number of people could get help from | Other | 0 (0.0) | 131 (82.9) | 0.0 | 0 (0.0) | 122 (80.8) | 0.0 |
|  | No people to one or more |  | 35 (22.2) | **0.90 (0.29, 1.52)** |  | 29 (19.2) | -2.66 (-6.00, 0.67) |
| NLES: change in knowing someone who had an accident | Other | 7 (4.2) | 134 (84.3) | 0.0 | 8 (5.3) | 123 (86.0) | 0.0 |
|  | From yes to no |  | 25 (15.7) | **1.28 (0.66, 1.90)** |  | 20 (14.0) | **7.86 (2.43, 13.29)** |
| NLES: change in drug and/or alcohol problems | Other | 7 (4.2) | 137 (86.2) | 0.0 | 8 (5.3) | 123 (86.0) | 0.0 |
|  | From yes to no |  | 22 (13.8) | 0.31 (-0.50, 1.11) |  | 20 (14.0) | **6.29 (0.45, 12.13)** |
| NLES: change in knowing someone sent to jail | Other | 7 (4.2) | 134 (84.3) | 0.0 | 8 (5.3) | 120 (83.9) | 0.0 |
|  | From yes to no |  | 25 (15.7) | **1.33 (0.68¸1.98)** |  | 23 (16.1) | 3.41 (-1.83, 8.65) |
| NLES: change in witness to violence | Other | 8 (4.8) | 126 (79.7) | 0.0 | 9 (6) | 112 (78.9) | 0.0 |
|  | From yes to no |  | 32 (20.3) | **0.84 (0.17, 1.50)** |  | 30 (21.1) | **5.63 (1.18, 10.08)** |
| NLES: change gambling problems | Other | 7 (4.2) | 123 (77.4) | 0.0 | 9 (6) | 112 (78.9) | 0.0 |
|  | From yes to no |  | 36 (22.6) | **0.55 (0.01, 1.09)** |  | 30 (21.1) | 2.53 (-1.67, 6.74) |
| NLES: change in number of stressors quartiles | 12 to 3 (improved) | 10 (6.0) | 41 (26.3) | 0.0 | 12 (8.0) | 39 (28.1) | 0.0 |
|  | 2 to 0 |  | 45 (28.8) | -0.62 (-1.37, 0.13) |  | 37 (26.6) | -2.90 (-7.15, 1.36) |
|  | -1 to -2 |  | 32 (20.5) | -0.33 (-1.02, 0.36) |  | 29 (20.9) | -3.82 (-9.51, 1.86) |
|  | -3 to -10 (worsened) |  | 38 (24.4) | **-1.25 (-1.88¸-0.62)** |  | 34 (24.5) | -4.29 (-8.73, 0.14) |
| NLES: number changing to positive | 0-1 | 15 (9.0) | 71 (47.0) | 0.0 | 12 (8.0) | 39 (28.1) | 0.0 |
|  | 2 |  | 24 (15.9) | 0.66 (-0.06, 1.38) |  | 37 (26.6) | 2.43 (-2.54, 7.40) |
|  | 3 |  | 23 (15.2) | **0.93 (0.14, 1.72)** |  | 29 (20.9) | 2.33 (-2.31, 6.97) |
|  | 4-10 |  | 33 (21.9) | **0.83 (0.14, 1.51)** |  | 34 (24.5) | 5.15 (0.09, 10.21) |
| **Child health and health related behaviour** |  |  |  |  |  |  |  |
| Change in number of illnesses amongst children | -11 to -2 (less illness in children) | 0 (0.0) | 43 (25.9) | 0.0 | 0 (0.0) | 39 (25.8) | 0.0 |
|  | -1 to 0 |  | 63 (38.0) | **-0.71 (-1.38, -0.03)** |  | 60 (39.7) | -0.18 (-4.43, 4.07) |
|  | 1 to 1 |  | 29 (17.5) | -0.75 (-1.55, 0.04) |  | 24 (15.9) | 0.13 (-5.68, 5.94) |
|  | 2 to 7 (more illness in children) |  | 31 (18.7) | -0.15 (-0.96, 0.67) |  | 28 (18.5) | -0.89 (-5.06, 3.29) |
| Change in Overall House SCS | Limited or no change | 26 (15.7) | 78 (55.7) | 0.0 | 23 (15.2) | 69 (53.9) | 0.0 |
|  | Deterioration of 2 or more |  | 28 (20.0) | 0.43 (-0.26, 1.12) |  | 27 (21.1) | 4.47 (-0.63, 9.58) |
|  | Improvement of 2 or more |  | 34 (24.3) | **-0.88 (-1.50, -0.26)** |  | 32 (25.0) | 0.61 (-2.66, 3.89) |

1 NegAB - Carer Negative affect balance (range, -4 to 4); 2 BSD – Carer Risk of depression (range, -30 to 30).

A positive Beta coefficient indicates the variable is associated with an improvement in mental health. Bold font indicates the variable was significant at p0.05; ns – not significant
